# Outcomes for primary kidney transplantation from donation after Citizens’ death in China: a single center experience of 367 cases

**DOI:** 10.1186/s12913-017-2190-7

**Published:** 2017-04-04

**Authors:** Wujun Xue, Puxun Tian, Heli Xiang, Xiaoming Ding, Xiaoming Pan, Hang Yan, Jun Hou, Xinshun Feng, Linjuan Liu, Chenguang Ding, Xiaohui Tian, Yang Li, Jin Zheng

**Affiliations:** Department of Renal Transplant, Center of Nephropathy, the First Affiliated Hospital of Xi’an Jiaotong University, Xi’an 710061, 277 Yanta West Road, Xi’an, 710061 China

**Keywords:** DBCD, DCD, Organ donation, DGF, Kidney transplantation

## Abstract

**Background:**

The cases of donation after brain death followed by circulatory death (DBCD) and donation after cardiac death (DCD) have been increased year by year in China. Further research is needed to understand in the outcomes and risk factors of delayed graft function (DGF) in order to minimize the risk of DGF and ameliorate its potential impact on long-term outcomes. This study was to explore the differences in outcomes between DBCD and DCD transplant and the main risk factors for DGF in DBCD.

**Methods:**

Retrospective analysis of the clinical data of 367donations after citizens’ death kidney transplant procedures (donors and recipients) between July 2012 and August 2015 at our center.

**Results:**

During the study period, the donation success rate was 25.3%. 164 cases of DBCD and 35 cases of DCD had been implemented and 367 kidneys were transplanted. The incidence of DGF in DBCD group were significantly lower than that of DCD group (12.0% vs. 27.0%, *p* = 0.002). The 1-year percent freedom from acute rejection (AR) was significantly higher in DBCD group compared with it of DCD group (94% vs. 82%, *p* = 0.036). Multivariate logistic regression analysis of the kidney transplants revealed that the high risk factors for DGF after renal transplantation in DBCD were history of hypertension (Odds Ratio [OR] = 5.88, 95% CI: 1.90 to 18.2, *p* = 0.002), low blood pressure (BP < 80 mmHg) (OR = 4.86, 95% CI: 1.58 to 14.9, *p* = 0.006) and serum creatinine of donor (OR = 1.09, 95% CI: 1.03 to 1.16, *p* = 0.003) before donation.

**Conclusions:**

The outcomes of DBCD could be better than DCD in DGF and AR. The main risk factors for DGF in DBCD kidney transplants are donors with a history of hypertension, low blood pressure, and serum creatinine of donor before donation.

## Background

Donation after cardiac death (DCD) and donation after brain death (DBD) are two processes for organ donation. Transplant technology and experiences with DCD and DBD resources are now highly advanced, and the transplantation outcomes of both organ resources processes are well explored [1]. Although DBD has been accepted in Western nations for a long time, a vast majority of people in the Chinese society have not fully accepted the Harvard brain death criteria [2], besides, the relevant laws on brain death have not yet to be approved in China [3]; thus, potential donors who meet the brain death criteria still have to wait for cardiac arrest before donation, which donation was named controlled DCD after brain death or donation after brain death followed by circulatory death (DBCD).

In 2012, Dr. Qiu and his colleagues summarized the introduction of DBCD in China as a new protocol for deceased donations, which responded to brain death not being legally recognized in China [4]. Additional recent studies of clinical outcomes have shown that pediatric DBCD kidney donation for transplant is also feasible [5]. Furthermore, the DBCD program adheres to the guiding principles of the World Health Organization, and is compliant with the Declaration of Istanbul. In 2012 and 2014, Professors Huang and Sun published China DBCD-related policies regarding the implementation of standards and specifications in The Lancet [3, 6].

Delayed graft function (DGF) is an early complication after deceased donor kidney transplantation with significant adverse effects on graft outcomes. One of the major concerns of kidneys from DCD donors is the high incidence of DGF observed after transplantation. The harmful impact of DGF on graft outcomes in DBD transplant is well documented [2, 7–9]. However, there was no related report on clinical outcomes of kidneys from DBCD donors.

In China, the cases of DBCD and DCD have been increased year by year. Further research is needed to understand in the outcomes and risk factors of DGF in the DBCD transplants, so that to minimize the risk of DGF and ameliorate its potential impact on long-term outcomes. The aim of this study was to compare the outcomes of DBCD transplants with those of DCD transplants, and explore the risk factors of DGF among patients with DBCD kidney transplantation.

## Methods

### Design

This was a single-center, retrospective cohort study conducted at the First Affiliated Hospital of Xi’an Jiaotong University, Xi’an, China. Data were obtained from the registry system of organ donation database, which collects all data prospectively. All patients who received a deceased-donor kidney-only transplant from July 2012 to August 2015 were included in this study. Recipients were regularly followed up post transplant. The follow up time points were the first month, the second months, the third month, the sixth month, 1 year after kidney transplantation, then once a year. The median follow up time was 317 days, range from 1 day to 700 days. Recipients were divided into two groups based on the type of donor DBCD and DBD. End points studied were patient survival and uncensored graft survival. Other end points included DGF, AR, and infection in the first year.

### Donors and recipients information

A Donation after Citizens’ Death flow chart for donors in China is briefly shown in our previous study [10]. Acceptable criteria for donors include: donor is identified; no history of kidney disease, drug abuse, uncontrolled hypertension, diabetes mellitus with complications, malignancy and systemic sepsis; no active HIV infection; donor age ≤ 65 years old; warm ischemia time ≤ 60 min (life-support withdrawal to aortic in situ cold perfusion). Kidneys of donation after citizens’ death were regionally distributed within the organ sharing network system of China, which is similar to the United Network for Organ Sharing.

Three hundred sixty seven kidney transplant cases derived from 199 donors were completed in our center, in accordance with the donation after citizens’ death standard implementation at our local organ procurement organization (OPO). The family members (parents, spouses, and adult children) of the donors agreed to the organ donation after cardiac death, and signed a voluntary organ donation form and other related documents. Organs were recovered after removal of the donor from mechanical ventilation and 2 ~ 5 min after confirmation of cardiac death. All kidneys were preserved by hypothermic machine perfusion (Life Port Kidney Transporter, Shanghai Genext Medical Technology Co., Ltd.). A kidney transplantation would not be performed if machine perfusion parameters showed that a flow rate < 80 mL/min and/or resistant index >0.4. The characteristics of the donors and recipients were reported in Tables 1 and 2.

**Table 1:** Characteristics of the donors (*N* = 199)^a^

Category	DBCD (*n* = 164)	DCD (*n* = 35)	*P*-value
Age (years)	39.4 ± 15.7	40.9 ± 17.6	0.599
Male, n (%)	130 (79.3%)	29 (82.9%)	0.631
BMI (kg/m^2^)	21.6 ± 3.1	21.0 ± 3.8	0.316
Reason for death, n (%)			
Craniocerebral injury	110 (67.1%)	10 (28.6%)	<0.001
Cerebral hemorrhage	39 (23.8%)	12 (34.3%)	
Anoxic encephalopathy	15 (9.2%)	13 (37.1%)	
Duration of ischemia			
Warm ischemia (min)	10.2 ± 7.0	10.4 ± 10.0	0.921
Cold ischemia (h)	4.66 ± 1.86	4.81 ± 2.26	0.698
Serum creatinine (μmol/L)	115.7 ± 80.6	94.6 ± 147	0.421
Vasopressors, n (%)	119 (75.6%)	20 (57.1%)	0.071
Mechanical ventilation, n (%)	162 (98.8%)	34 (97.1)	0.442
History of Hypertension	27 (16.5%)	9 (25.7%)	0.197
History of Diabetes	3 (1.83%)	2 (5.71%)	0.213
Hypotension			
BP^§^ <100 mmHg	37 (22.6%)	5 (14.3%)	0.276
BP^§^ <80 mmHg	18 (11.0%)	2 (5.71%)	0.537
BP^§^ <50 mmHg	5 (3.05%)	4 (11.4%)	0.053
CPR			
Conducted CPR?	25 (15.2%)	5 (14.3%)	0.886
Duration of CPR (min)	13.6 ± 9.6	41.2 ± 44.9	0.242

^a^Continuous variables were reported as means ± standard deviations; categorical variables were reported as frequencies (percentages)

^§^ *BP* blood pressure

**Table 2:** Characteristics of the recipients (*N* = 367)^a^

Category	DBCD (*n* = 303)	DCD (*n* = 64)	*P*-value
Male, n (%)	209 (69.0%)	39 (60.9%)	0.212
Age (years)	36.3 ± 10.5	35.9 ± 10.3	0.752
BMI (kg/m^2^)	20.7 ± 3.2	19.8 ± 3.3	0.026
Primary diseases, n (%)			
Chronic glomerulonephropathy	230 (75.9%)	47 (73.4%)	0.963
gA nephropathy	27 (8.9%)	7 (10.9%)	
Diabetic nephropathy	18 (5.9%)	4 (6.3%)	
Others	28 (9.2%)	6 (9.4%)	
Negative PRA, n (%)	284 (93.7%)	57 (89.1%)	0.186
HLA mismatches	2.31 ± 0.84	2.20 ± 0.78	0.347
Dialysis, n (%)			
Hemodialysis vs. Peritoneal dialysis	280 (92.4%)	58 (90.6%)	0.631
Dialysis duration (days)	242 ± 218	225 ± 209	0.584

^a^Continuous variables were reported as means ± standard deviations; categorical variables were reported as frequencies (percentages)

### HLA typing and epitope mismatch identification

High-resolution HLA typing (HLA-A, −B,-C, −DR, and -DQ) was performed using sequence-specific oligo nucleotide probes (LAB Type_HD SSO, One Lambda, Canoga Park, CA, USA). HLA types A, B, and DR (three pairs and six antigens) are used for matching before kidney transplantation. All of the recipients were complement-dependent-cytotoxicity-crossmatch negative and therefore considered to be at low risk of early AR.

### Immunosuppression regimen and prophylactic treatment

All of the recipients were given a triple immunosuppressive regimen with calcineurin inhibitors (CNIs), enteric-coated mycophenolate sodium (EC-MPS; Myfortic, Novartis Pharma, Basel, Switzerland), and prednisone. The CNIs included cyclosporine A (CsA; Sandimmun Optoral, Novartis Pharma, Nuremberg, Germany) and tacrolimus (TAC; Prograf, Astellas Pharma, Deerfield, IL, USA). The initial dosages of CsA, TAC, EC-MPS, and prednisone were 4.0-4.5 mg/kg/day, 0.06-0.08 mg/kg/day, 1080–1440 mg/day, and 10–20 mg/day, respectively. All of the recipients were treated with rabbit anti-thymocyte globulin (rATG; thymoglobulin, Genzyme Ireland, Waterford, Ireland) at a dosage of 1–1.25 mg/kg/day as induction therapy during the surgery, and for a total of 4–6 days after kidney transplantation.

Anti-infective prophylaxis included oral intake of sulfamethoxazole/trimethoprim for 6 months and intravenous administration of valganciclovir for 2 weeks, which was initiated immediately and after 2 months post-transplantation, respectively. This was followed by maintenance therapy with oral ganciclovir for at least 3 months, depending on donor and recipient cytomegalovirus (CMV) serological status.

### Definitions

DGF was defined as a serum creatinine concentration at postoperative day 7 of >2.5 mg/dL or the need for dialysis during the first week after transplantation.

AR was identified on biopsy and classified according to the Banff’07 classification and its subsequent updates [11]. AR was suggested clinically by an unexplained rise in serum creatinine concentration of >0.3 mg/dL or a 25% increase from baseline, and was confirmed by ultrasound-guided percutaneous biopsy. The incidence, time, and therapy for AR were noted within 12 months after transplantation.

The infections within the first year of kidney transplantation were also explored in this study, including pulmonary infection, urinary tract infection, and incision infection.

### Post-transplantation monitoring

Serum concentrations of total bilirubin, alanine aminotransferase, albumin, blood urea nitrogen, and serum creatinine were measured using an automatic biochemistry analyzer (7170, Hitachi, Tokyo, Japan). The blood trough concentrations of CsA, TAC and Mycophenolic acid (MPA) were measured regularly using an enzyme multiplied immunoassay (Siemens Healthcare Diagnostic, Camberley, UK). The PRA and donor-specific antibody (DSA) were screened using the Luminex 200 system (Luminex, Austin, TX, USA).

### Statistical analysis

Data were analyzed by SPSS® version 17.0. Categorical data were compared using the Chi-square tests or Fisher’s exact tests, while continuous data were analyzed using Mann–Whitney *U* test or Student’s *t* test, as appropriate. Kaplan-Meier non-parametric methods were used to determine the one-year freedom from the events (death, AR and infection) after kidney transplantation. Log rank tests were used to compare the difference in them between DBCD and DCD patients. Univariate and multivariate logistic regression analyses were performed to identify risk factors of DGF in the DBCD group. Variables with P values <0.1 in the univariate analyses were entered into the multivariate logistic regression models. Odds ratios (ORs) were presented with their 95% CIs. *P* < 0.05 was considered statistically significant.

## Results

### Characteristics of donors and recipients

Among the 786 families in our study, 587 families did not give consent to donate organs of potential donors, while the remaining 199 families provided consent. Donation success rate was 25.3%. The conditions of 164 (82.4%) donors complied with the standard of DBCD. The other 35 cases were complied with DCD. The average ages of the donors and recipients were 39.6 ± 16.1 and 36.2 ± 10.5 years, respectively. There are 159 (79.9%) male out of 199 donors and 248 (67.6%) male out of 367 recipients. A comparison of donors and recipients characteristics between DBCD patients and DCD patients is displayed in Tables 1 and 2. Donors in DBCD group had higher craniocerebral injury rate for death cause (67.1% vs. 28.6%, *p* < 0.001) and BMI of recipients was a little higher in DBCD group compare it of DCD group (20.7 ± 3.2 vs. 19.8 ± 3.3, *p* = 0.029). Other clinical characteristics were not found significantly different between DBCD and DCD groups.

### Outcome of transplantation

**Fig. 1:**
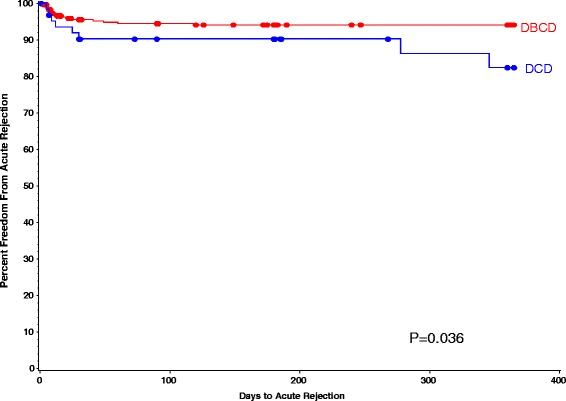
The one-year Percent freedom from acute rejection in DBCD and DCD group

**Fig. 2:**
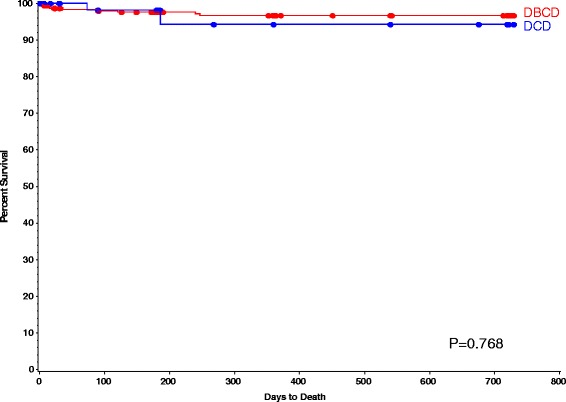
The one-year patients survival rate of DBCD and DCD group

**Fig. 3:**
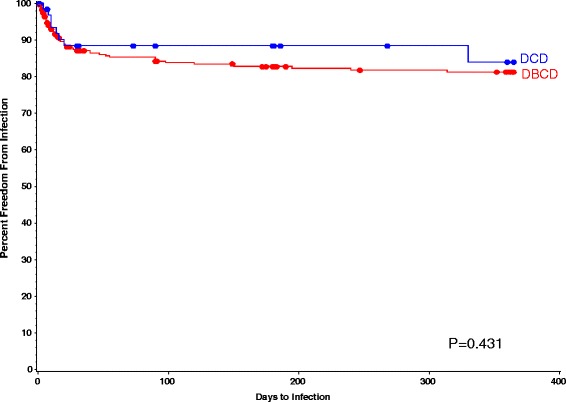
The one-year percent freedom from infection in DBCD and DCD group

As described in Table 3, the incidence of DGF in DBCD group were significantly lower than that of DCD group (12.0% vs. 27.0%, *p* = 0.002), and one-year percent freedom from AR was higher in DBCD group compared with it of DCD group (94% vs. 82%, *p* = 0.036), respectively (Fig. 1). Those differences stayed significant after the death cause of donor and BMI of recipient were adjusted. The 1-year patients survival rate of DBCD and DCD group was 97% and 94% (*p* = 0.768) (Fig. 2). No different between the two groups were found on graft excision rates (DBCD 3.3% vs. DCD4.7%, *p* = 0.707), and one-year percent freedom from infection (DBCD 85% vs. DCD 88%, *p* = 0.431) (Fig. 3).

**Table 3:** Events After Kidney Transplantation By Group^a^

Category	DBCD (*n* = 303)	DCD (*n* = 64)	*P*-value
Delayed graft function, n (%)	36 (12.0%)	17 (27.0%)	0.002
Graft excision, n (%)	10 (3.3%)	3 (4.7%)	0.707
Survival	97%	94%	0.768
Acute rejection	94%	82%	0.036
Any infection	85%	88%	0.431

^a^Delayed graft function and graft excision were reported as frequencies (percentages); the rest outcomes were reported as 1-year percent freedom from events, Kaplan-Meier method was used to estimated the 1-year percent freedom from the events and log rank tests were used to compare the events between DBCD and DCD

### Risk factors of DGF occurrence within DBCD

Results of univariate analyses for the risk factors of DGF were reported in Table 4. Within the DBCD group, recipients with donors died from cerebral hemorrhage (OR: 2.27, 95%CI: 1.03 to 5.00, *p* = 0.041) and other diseases (OR: 3.18, 95% CI: 1.13 to 8.94, *p* = 0.028) had higher risk of developing DGF compared with the recipients whose donors died from craniocerebral injury. Higher serum creatinine of donor’s (OR = 1. 10, 95% CI:1.06 to1.14, p < 0.001) before donation, longer cold ischemic time (OR = 1.21, 95% CI, 1.02 to1.44, *p* = 0.033), processes of CPR (OR = 3.95, 95% CI: 1.79 to 8.71, *p* = 0.001), history of hypertension (OR = 4.85, 95% CI: 2.25 to 10.4, *p* < 0.001) for donors were found associated with higher risk of DGF. Donors experienced in hypotension would increase the risk of DGF for recipients (BP < 100 mmHg: [OR = 3.53, 95% CI: 1.72 to 7.25, *p* = 0.001]; BP < 80 mmHg [OR = 5.16, 95% CI: 2.23 to 11.9, p < 0.001]; BP < 50 mmHg [OR = 8.00, 95% CI: 1.55 to 41.3, *p* = 0.013). If a donor used vasopressor agents, the recipient of the kidney would have lower risk of DGF after the kidney transplantation (OR: 0.37, 95% CI: 0.18 to 0.76, *p* = 0.007).

**Table 4:** Univariate Analysis for Risk factors of DGF in DBCD group (*N* = 303)^a^

Risk factors		Odds ratios	95% confidence interval	*P* value
Age, years		0.98	0.96 to 1.01	0.153
Male		0.32	0.15 to 0.65	0.002
BMI		1.03	0.92 to 1.16	0.610
Reason for brain death	Craniocerebral injury	Reference group		
	Cerebral hemorrhage	2.27	1.03 to 5.00	0.041
	Other	3.18	1.13 to 8.94	0.028
Serum creatinine (μmol/L)		1.10	1.06 to 1.14	<0.001
Warm ischemia, minutes		0.99	0.94 to 1.05	0.816
Cold ischemic time, minutes		1.21	1.02 to 1.44	0.033
CPR conducted		3.95	1.79 to 8.71	0.001
Vasopressor agents used		0.37	0.18 to 0.76	0.007
Had Hypertension		4.85	2.25 to 10.4	<0.001
Hypotension (BP < 100 mmHg)		3.53	1.72 to 7.25	0.001
Hypotension (BP < 80 mmHg)		5.16	2.23 to 11.9	<0.001
Hypotension (BP < 50 mmHg)		8.00	1.55 to 41.3	0.013

^a^Logistic regressions were performed, odds ratios and the 95% confidence intervals were reported

In multivariate logistic regression analysis, history of hypertension (OR = 5.88, 95% CI: 1.90 to 18.2, *p* = 0.002), hypotension (BP < 80 mmHg [OR = 4.86, 95% CI: 1.58 to 14.9, *p* = 0.006]), and serum creatinine of donors before donation (OR = 1.09, 95% CI: 1.03 to 1.16, *p* = 0.003) were risk factors of DGF occurrence (Table 5). Vasopressor agents (OR = 0.27, 95% CI: 0.11 to 0.71, *p* = 0.008) was benefits factor for DGF in our study.

**Table 5:** Multivariate Analysis for Risk factors of DGF in DBCD group (*N* = 303)^a^

Risk Factors		Odds ratios	95% confidence interval	*P* value
Death cause	Craniocerebral injury	Reference Group		
	Cerebral hemorrhage	0.79	0.27 to 2.35	0.672
	Other	2.86	0.62 to 13.2	0.178
Serum Creatinine, per 10 μmol/L		1.09	1.03 to 1.16	0.003
Cold ischemic time, minutes		1.12	0.88 to 1.43	0.345
CPR conducted		0.99	0.26 to 3.77	0.985
Vasopressor agents used		0.27	0.11 to 0.71	0.008
Had Hypertension		5.88	1.90 to 18.2	0.002
Hypotension (BP < 80 mmHg)		4.86	1.58 to 14.9	0.006

^a^Logistic regressions were performed, odds ratios and the 95% confidence intervals were reported

## Discussion

The DBCD donation cases in our study are cases of waiting cardiac or circulatory death includes patients for whom circulatory death occurs after a planned withdrawal of life-sustaining therapies. The DCD donation cases of ours include sudden unexpected circulatory arrest without any attempt of resuscitation, sudden unexpected irreversible circulatory arrest with unsuccessful resuscitation, and sudden circulatory arrest after brain death diagnosis. Actually, the definition of Chinese term “DBCD” and DCD are identical with some contents of “Controlled DCD” and “Uncontrolled DCD” as described by Marie Thuong, et al. in 2016 [12, 13]. In Table 6, Organ donation in China was classified into the Maastricht Classification of DCD according to the modified Maastricht Classification of DCD [12].

**Table 6:** The Modified Maastricht Classification and China Classification of DCD

	Maastricht classification	China classification
Category I Uncontrolled	Found dead IA. Out-of-hospital IB. In-hospital	II Organ donation after cardiac death (DCD)^a^
Category II Uncontrolled	Witnessed cardiac arrest IIA. Out-of-hospital IIB. In-hospital	II Organ donation after cardiac death (DCD)^a^
Category III Controlled	Withdrawal of life-sustaining therapy	III Organ donation after brain death followed by circulatory death (DBCD)
Category VI Uncontrolled Controlled	Cardiac arrest while life brain dead	II Organ donation after cardiac death (DCD)

^a^All the cases of DCD in our study were in–hospital

The research data of DCD renal transplantations carried out in Europe and reported by Moers, Kokkinos, and Jochmans show that the rate of DGF in DBD and DCD transplants were 13–35 and 28–88% respectively [14–16]. In 2011, the data from Wake Forest University revealed that the incidence of AR after kidney transplantation was 28.6% for DCD kidneys and 16.1% for DBD kidneys. The incidence rates of infection after renal transplantation for DCD and DBD were 28.5 and 26.1%, respectively [17, 18].

In our study, we compared the outcome of 64 DCD and 303 DBCD kidney transplant. Our results indicated the effect of DBCD was better than DCD in DGF and AR. The various indexes of donors and recipients including demography characteristics, index of renal function before donation, history of primary disease, HLA mismatches and dialysis were similar between DBCD and DCD groups. DBCD in our study is belonging to controlled DCD, while DCD is belonging to uncontrolled DCD. Therefore, it is easy to understand why the overall outcomes of DBCD are close to that for DBD and better than that for DCD. These data reflected that the real reason decided the good transplant effect was the processes of donation.

The major underlying mechanism of DGF is related to ischaemia/reperfusion injury, which includes microvascular inflammation and cell death and apoptosis, and to the regeneration processes. Clinical factors related to donor, recipient and organ procurement/transplantation procedures may increase the risk of DGF. Some of these parameters have been used in specific predictive formulas created to assess the risk of DGF [19]. The important finding of this study for the high risk factors of DGF after renal transplantation were history of hypertension, serum creatinine of donor before donation, and low blood pressure (BP < 80 mmHg).

The incidence of DGF after renal transplantation in the present study was 27% in recipients whose grafts were recovered from donors with a history of hypertension of at least 5 years. For such donors, it is necessary to learn more about their history of anti hypertensive treatment and treatment outcomes, whether they suffered complications of hypertension, and its influence on renal function. The donor’s final serum creatinine value was one of important proposed risk factors for DGF [20]. Multivariate logistic regression analysis in our study also showed that higher serum creatinine of donors (OR = 1.09, [95% CI, 1.03 to 1.16], *p* = 0.003) before donation was risk factors of DGF occurrence, which was identical with others reports.

Kidney is an organ of hypertransfusion and very sensitive to ischemia, while Low blood pressure results the insufficient of kidney blood flow volume, which will induce ischemia/reperfusion injury on kidney and clinically present as the DGF. In our study, the data of univariate analysis showed that donors experienced in hypotension (BP < 100 mmHg) would increase the risk of DGF for recipients. However, multivariate analysis data showed hypotension experience with blood pressure less than 80 mmHg became significant after all variables with *P* < 0.1 in the univariate analyses were included in one multivariate model, it could be due to lack of power. In brief, both univariate and multivariate analysis showed the lower of the blood pressure, the higher of the DGF risk. The vasopressor agents used in our study were vascular active pharmaceutical (dopamine, aramine, norepinephrine) to stabilize the donor’s blood pressure. All of the pharmaceuticals have minimal impact on renal function. On the other hand, the vasopressor agents ensure the sufficient blood in organs before donation, which is benefit for the function recovery of graft after transplantation.

It was showed that cold ischemia time has a critical effect on the development of DGF. The two most widely used preservation buffers, University of Wisconsin (UW) and histidine-tryptophan-ketoglutarate (HTK) solutions, show similar efficacy in terms of DGF rates for most donor types [21, 22]. Machine perfusion was introduced in the early days of solid organ transplantation to minimize the adverse effects of cold storage on retrieved organs. Lifeport has the role of evaluating the quality of kidney, removing residual blood clots, reducing perfusion resistance, protecting the kidney, so that reducing the occurrence of DGF [7, 23].

Limitations of our study include, first, it’s a retrospective study and some information of donors and recipients are collected incompletely, which could influence the occurrence of DGF and graft outcomes. Second, a variety of other clinical factors may also increase the risk of DGF, such as marked body mass index of the donor and recipient, potential drug nephrotoxicity, surgical problems and/ or hyperimmunization of the recipient [19]. It should be detected any significant differences in outcomes in DBCD recipients with and without DGF. Finally, DGF may decrease the long-term graft function, but reports on this effect are inconsistent [9, 19]. So the longer-term impact of DGF in recipients of DBCD and DCD kidney transplants needs to be seen.

## Conclusions

In conclusion, the clinical outcomes of DBCD kidney transplantation is better than DCD kidney transplantation. Successful implementation of DBCD and subsequent kidney transplantation requires an accurate and timely assessment and maintenance of the function of the donated organs. The main risk factors of DGF are donors who have a history of hypertension, serum creatinine of donor before donation, and low blood pressure of donor before donation.

## Abbreviation

AR: Acute rejection
BMI: Body mass index
CNIs: Calcineurin inhibitors
CPR: Cardiopulmonary resuscitation
CsA: Cyclosporine A
DBCD: Donation after brain death followed by circulatory death
DBD: Donation after brain death
DCD: Donation after cardiac death
DGF: Delayed graft function
DSA: Donor-specific antibody
EC-MPS: Enteric-coated mycophenolate sodium
HLA: Human leukocycte antigen
MPA: Mycophenolic acid
OPO: Organ procurement organization
PRA: Panel reactive antibody
rATG: Rabbit antithymocyte globulin
TAC: Tacrolimus

## References

[1] Allen AM, Kim WR, Xiong H, Liu J, Stock PG, Lake JR, Chinnakotla S, Snyder JJ, Israni AK, Kasiske BL (2014). Survival of recipients of livers from donation after circulatory death who are relisted and undergo retransplant for graft failure. Am J Transplant.

[2] Sudhindran S, Pettigrew GJ, Drain A, Shrotri M, Watson CJ, Jamieson NV, Bradley JA (2003). Outcome of transplantation using kidneys from controlled (Maastricht category 3) non-heart-beating donors. Clin Transpl.

[3] Huang J, Millis JM, Mao Y, Millis MA, Sang X, Zhong S (2012). A pilot programme of organ donation after cardiac death in China. Lancet.

[4] Qiu ZQ, Tan WF, Yan PN, Luo XJ, Zhang BH, Wu MC, Jiang XQ, Lau WY (2012). Early control of short hepatic portal veins in isolated or combined hepatic caudate lobectomy. Hepatobiliary Pancreat Dis Int.

[5] Li JF, Liu J, Guo T, Pang XL, Liu L, Feng YH, Wang ZG, Feng GW, Shang WJ (2014). Kidney transplantation from pediatric donors in a single Chinese center. Cell Biochem Biophys.

[6] Sun Q, Gao X, Wang H, Ko DS, Li XC (2014). A new era for organ transplantation in China. Lancet.

[7] Chamorro C, Falcon JA, Michelena JC (2009). Controversial points in organ donor management. Transplant Proc.

[8] Snoeijs MG, Winkens B, Heemskerk MB, Hoitsma AJ, Christiaans MH, Buurman WA, van Heurn LW (2010). Kidney transplantation from donors after cardiac death: a 25-year experience. Transplantation.

[9] Nagaraja P, Roberts GW, Stephens M, Horvath S, Fialova J, Chavez R, Asderakis A, Kaposztas Z (2012). Influence of delayed graft function and acute rejection on outcomes after kidney transplantation from donors after cardiac death. Transplantation.

[10] Xiaoming P, Xiang H, LinJuan L, Chenguang D, Ren L (2015). Preliminary results of transplantation with kidneys donated after cardiac death: a path of hope for organ transplantation in China. Nephrol Dial Transplant.

[11] Solez K, Colvin RB, Racusen LC, Haas M, Sis B, Mengel M, Halloran PF, Baldwin W, Banfi G, Collins AB (2008). Banff 07 classification of renal allograft pathology: updates and future directions. Am J Transplant.

[12] WHO guiding principles on human cell, tissue and organ transplantation. Transplantation 2010, 90(3):229–233. 10.1097/TP.0b013e3181ec29f0 20664493

[13] Thuong M, Ruiz A, Evrard P, Kuiper M, Boffa C, Akhtar MZ, Neuberger J, Ploeg R (2016). New classification of donation after circulatory death donors definitions and terminology. Transpl Int.

[14] Moers C, Leuvenink HG, Ploeg RJ (2007). Non-heart beating organ donation: overview and future perspectives. Transpl Int.

[15] Kokkinos C, Antcliffe D, Nanidis T, Darzi AW, Tekkis P, Papalois V (2007). Outcome of kidney transplantation from nonheart-beating versus heart-beating cadaveric donors. Transplantation.

[16] Jochmans I, Darius T, Kuypers D, Monbaliu D, Goffin E, Mourad M, Ledinh H, Weekers L, Peeters P, Randon C (2012). Kidney donation after circulatory death in a country with a high number of brain dead donors: 10-year experience in Belgium. Transpl Int.

[17] Wadei HM, Heckman MG, Rawal B, Taner CB, Farahat W, Nur L, Mai ML, Prendergast M, Gonwa TA (2013). Comparison of kidney function between donation after cardiac death and donation after brain death kidney transplantation. Transplantation.

[18] Singh RP, Farney AC, Rogers J, Zuckerman J, Reeves-Daniel A, Hartmann E, Iskandar S, Adams P, Stratta RJ (2011). Kidney transplantation from donation after cardiac death donors: lack of impact of delayed graft function on post-transplant outcomes. Clin Transpl.

[19] Grenda R. Delayed graft function and its management in children. Pediatr Nephrol. 2016;1-11. 10.1007/s00467-016-3528-9 27778091

[20] Björn N, Mario AF, Franco C: Prediction, prevention, and management of delayed graft function: where are we now? Clinical transplantation 2016:1–11. 10.1111/ctr.12832 27543840

[21] O'Callaghan JM, Knight SR, Morgan RD, Morris PJ (2012). Preservation solutions for static cold storage of kidney allografts: a systematic review and meta-analysis. Am J Transplant.

[22] de Boer J, De Meester J, Smits JM, Groenewoud AF, Bok A, van der Velde O, Doxiadis II, Persijn GG (1999). Eurotransplant randomized multicenter kidney graft preservation study comparing HTK with UW and Euro-Collins. Transpl Int.

[23] Lodhi SA, Lamb KE, Uddin I, Meier-Kriesche HU (2012). Pulsatile pump decreases risk of delayed graft function in kidneys donated after cardiac death. Am J Transplant.

